# Effects of Medical Insurance on the Health Status and Life Satisfaction of the Elderly

**Published:** 2017-09

**Authors:** Liubao GU, Huihui FENG, Jian JIN

**Affiliations:** Dept. of Statistics, School of Economics, Hebei University, Baoding, China

**Keywords:** Health status, Life satisfaction, Medical insurance, Ordered probit model

## Abstract

**Background:**

Population aging has become increasingly serious in China. The demand for medical insurance of the elderly is increasing, and their health status and life satisfaction are becoming significant issues. This study investigates the effects of medical insurance on the health status and life satisfaction of the elderly.

**Methods:**

The national baseline survey data of the China Health and Retirement Longitudinal Survey in 2013 were adopted. The Ordered Probit Model was established. The effects of the medical insurance for urban employees, medical insurance for urban residents, and new rural cooperative medical insurance on the health status and life satisfaction of the elderly were investigated.

**Results:**

Medical insurance could facilitate the improvement of the health status and life satisfaction of the elderly. Accordingly, the health status and life satisfaction of the elderly who have medical insurance for urban residents improved significantly. The regression coefficients were 0.348 and 0.307. The corresponding regression coefficients of the medical insurance for urban employees were 0.189 and 0.236. The regression coefficients of the new rural cooperative medical insurance were 0.170 and 0.188.

**Conclusion:**

Medical insurance can significantly improve the health status and life satisfaction of the elderly. This development is of immense significance for the formulation of equal medical security.

## Introduction

The improvement of the economic development level of China has resulted in the enhancement of living conditions in the country, increase in life expectancy of citizens, and the emergence of the phenomenon of population aging (1). By the end of 2014, China’s “over the age of 60” population had reached 212,000,000, thereby accounting for 15.5% of the country’s total population. The China Urban Development Report (2015) predicted that China’s elderly population would reach 483,000,000 (i.e., one elderly for every three people) by 2050; this number would account for 34.1% of the total population. Thus, the studies on the health status and life satisfaction of the elderly have gradually focused on the issues related to the government and society.

Given this background of a severe population-aging problem, the elderly who are vulnerable to diseases (2) also have an increasing demand for medical insurance. The Chinese government is committed to achieve the medical and health services development goal of having “universal access to basic health care services” (3). The basic medical insurance systems for urban workers and residents in China were fully implemented in 1998 and 2010, respectively. All county-level administrative regions were included in the new rural cooperative medical care system in 2012. The integration of the basic medical insurance system for urban residents and the new rural cooperative medical care system has been enhanced since 2016. Accordingly, the Chinese government is establishing a unified urban and rural resident medical insurance system.

The medical insurance system (4) is an important component of social security systems. The former can provide fair and proper medical services or compensation for the people through the country or society to promote health and life satisfaction (5). Accordingly, this question should be answered: Has the medical insurance system in China, composed of the medical insurance of urban employees, medical insurance of urban residents and new rural cooperative medical service, achieved its desired purpose? The present study investigates this question with the elderly as the study object. Whether the medical insurance system in China has improved the living conditions and life satisfaction of the elderly was explored to provide a decision-making reference for the further improvement of the medical insurance system in China, as well as to offer new direction for the study of the elderly.

The microcosmic survey data of 4156 cases of the elderly over 60 years old were selected based on the nationwide baseline survey of the China Health and Retirement Longitudinal Survey in 2013. The effects of the medical insurance on the health (6–7) and life satisfaction were tested using the Ordered Probit Regression (8).

### Literature review

The elderly population cannot be ignored and needs the attention of the state and society (9). Moreover, the health and life satisfaction of the elderly have become a significant issue (10). The study on the effects of medical insurance on the health status and life satisfaction of the elderly facilitates the provision of a reference for perfecting the medical insurance system by focusing on the elderly.

Many scholars have conducted research on the relationship between medical insurance and health status.

A few scholars have reported that medical insurance brings a positive impact on health status. Xinjun Wang et al. empirically tested the effect of medical insurance on elderly health using the two-phase panel data of a Chinese longitudinal health longevity survey, as well as the panel logit and ordered models (11). Xinting et al. tested the effect of medical insurance on elderly health status using the binary logistic regression model. Their research showed that elderly health was divided into the physical and mental health (12). Finkelstein et al. investigated the medical service provided to Americans over 65 years old. Their result showed that the increase of the medical insurance coverage could improve medical service utilization and the self-rated health level and lower medical expenses (13). Pan et al. (14) used a nationwide panel data and confirmed the improvement of the health status of residents who availed of the medical insurance for urban residents. Qin et al. (15) used the instrumental variables estimation method as basis to determine that the medical insurance of urban employees could promote the self-rated health of rural migrant workers. A high health insurance coverage rate could improve public health, particularly for the poor (16). Courtemanche et al. (17) used the data from the behavioral risk factor monitoring system to confirm that the universal health insurance could improve the self-rated health in Massachusetts.

However, a few studies determined that health insurance lacks a positive effect on health status. The new rural cooperative medical insurance does not affect the mortality of children and pregnant women (18). A universal health insurance did not improve self-rated health with the triple difference method (19). Likwang et al. investigated the longitudinal survey data using the difference-in-differences method and determined that the increase of the medical service utilization rate brought by the universal health insurance did not decrease mortality or improve the self-rated health status (20).

For the influence of the medical insurance on the life satisfaction of the elderly, Shouwei et al. adopted the microcosmic survey data and tested the effects of the income level, health status, and medical insurance on the subjective well-being of 2200 people aged over 55 years using the ordered logit regression (21). Xiaomin validated the positive effects of the three types of social support on the life satisfaction of the elderly people based on the elderly population situation and willingness survey in Shanghai in 2013 through multiple linear regressions (22). Hongshu et al. adopted the China elderly health and longevity survey data in 2005 and investigated the effects of income inequality and health on the subjective well-being of the elderly in China. The result determined that the elderly of different income levels had different subjective well-being. Moreover, having medical insurance influences the subjective well-being of the elderly. The elderly with medical insurance had higher subjective well-being than those without medical insurance (23). Liao Pei An et al. adopted the health and living condition survey data and the triple difference method to investigate the effect of the Taiwan universal health insurance on the life satisfaction of the elderly. The result showed that the influences of a national health insurance on the life satisfaction of the elderly differed among genders. Accordingly, the female elderly were influenced more than the male elderly (24). A universal medical insurance significantly influenced well-being and life satisfaction, and the scope of influence ranged from 3% to 30% based on well-being (25). A universal health insurance improved the life satisfaction of older adults in Taiwan, particularly those who lack medical insurance (26). The increased coverage of medical insurance could improve the health and life quality of South Korean adults (27).

These studies have considered that the medical insurance could improve the life satisfaction of people. The subjective well-being defined happiness from cognition and emotion (28). Moreover, happiness is life satisfaction at the cognitive level, that is, the comprehensive evaluation of living quality is based on their own standards and expectations (29). Therefore, life satisfaction can be understood as a component of subjective well-being.

Existing research has focused on the impact of different types of medical insurance on public health status or the impact of the overall level of medical insurance on the life satisfaction of the elderly. This study aims to investigate the effects of medical insurance, as well as the different types of medical insurance, on the health status and life satisfaction of the elderly. Our study and previous studies are different from one another because the influences of medical insurance on the health status and life satisfaction of the elderly are given attention simultaneously. In addition, the current situation of the medical insurance system in China shows that medical insurance is subdivided into medical insurance for urban workers and urban residents, as well as the new rural cooperative medical system. Accordingly, their respective influences were explored.

This study investigated the effects of medical insurance on the health status and life satisfaction of the elderly.

## Methods

### Data resources and description Data resources

The data were derived from the China Health and Retirement Longitudinal Survey (CHARLS), which was a large-scale interdisciplinary survey project that collected the data of families and individuals aged over 45 years in China and conducted in 150 counties and 450 communities (villages) in 28 provinces (autonomous regions and municipalities). Multi-stage sampling was adopted in the project sampling with unequal probabilities in counties, districts, and villages. The research content was extensive and included basic information, family structure, financial support, health status, physical measurement, medical service utilization, medical insurance, work, retirement and pensions, income, consumption, assets, and basic situation of communities. Therefore, CHARLS can reflect the medical status, health status, and life satisfaction of the elderly in China. The data were derived from the 2013 CHARLS including 18605 sample units. A total of 8637 sample units were obtained after the missing data because various causes were excluded. The sample units aged 60 yr old and above were selected as the research objects following the definition of the WHO. The data of those aged 60 years old and above were separated. The final 4156 respondents were included in this study.

### Data description

The two variables explained in this study’s models are health status and life satisfaction. Health status was measured using self-rated health (30–31). Self-rated health was divided into five levels (i.e., 1–5), which represent very poor, poor, ordinary, good, and very good. The higher the score is, the better the health status becomes. The question in CHARLS for the dependent variable, i.e., life satisfaction (32–33), was “Overall, are you satisfied with your life?” The answer options were “extremely satisfied,” “very satisfied,” “satisfactory,” “unsatisfactory,” and “not satisfied.” This study used 1–5 to represent “not satisfied,” “unsatisfactory,” “satisfactory,” “very satisfied,” and “extremely satisfied,” respectively. The higher the score was, the higher the degree of satisfaction becomes.

The most important explanatory variable was medical insurance. CHARLS had a question on medical insurance: “Do you have medical insurance?”

The response options include without insurance, medical insurance for urban workers, medical insurance for urban residents, and new rural cooperative medical insurance. A few people selected commercial insurance. Thus, they were excluded in the models of this study. The three most popular responses, namely, medical insurance, medical insurance for urban workers, medical insurance for urban residents, and new rural cooperative medical insurance, were selected as the objects in this study.

Other variables included gender, age, type of registered permanent residence, marital status, education level, nationality, relative income, chronic disease (34), smoking, alcohol consumption, and leisure activities. Table 1 shows the definitions of the relevant variables.

**Table 1:** Variable definition

**Variable**	**Definition**
Medical insurance	Medical insurance=0(without any medical insurance), medical insurance=1(the medical insurance for urban workers), medical insurance=2(the medical insurance for urban residents), medical insurance=3(new rural cooperative medical insurance)
Health status	Health status=1 (very poor), health status=2 (poor), health status=3 (general), health status=4 (good), health status=5 (very good)
Life satisfaction	Life satisfaction =1(very dissatisfied), life satisfaction =2(not very satisfied), life satisfaction =3(general), life satisfaction =4(satisfied), life satisfaction =5(very satisfied)
Gender	Gender=1(male), gender=2(female)
Age	Age
Type of registered permanent residence	Type of registered permanent residence=1(agriculture account), type of registered permanent residence=2(non-agriculture account), type of registered permanent residence=3(Unified residence)
Marital status	People with spouse=1, people without spouse(single, divorced, bereft of one’s spouse)=0
Education level	Education level =1 (Primary education or lower), education level =2 (junior high school degree), education level =3 (high school degree or equivalent), education level =4 (college degree or higher)
Nationality	Han population=1, other nations=0
Relative income	Relative income=1 (much lower), relative income=2 (a little lower), relative income=3 (almost), relative income=4 (a little well), relative income=5 (very well)
Number of chronic disease	The universal chronic diseases of elderly include hypertension, diabetes, heart disease, stroke, asthma, lung diseases and cancer
Smoker or non-smoker	Smoker=1, non-smoker=0
Drinker or non-drinker	Drinker=1, non-drinker=0
Number of leisure activities	The number of the leisure activities of elderly

### Data statistics

Table 2 shows the descriptive statistics of each variable. Moreover, Table 2 shows that among 4156 samples used in this study, 166 people did not select any medical insurance, 483 people opted for medical insurance for urban workers, 210 people opted for medical insurance for urban residents, and 3297 selected new rural cooperative medical insurance. The respondents are between 60 and 95 years old and with an average 67.8 years old.

**Table 2:** Descriptive statistical analysis of variables

**Variable**	**Number of observations**			**Mean**	**Standard deviation**	**Minimum**	**Maximum**
Medical insurance	4156	Without any medical insurance	166	2.60	0.84	0	3
		Medical insurance for urban workers	483				
		Medical insurance for urban residents	210				
		New rural cooperative medical insurance	3297				
Health status		4156		2.91	0.93	1	5
Life satisfaction		4156		3.11	0.75	1	5
Gender		4156		1.64	0.48	1	2
Age		4156		67.80	6.60	60	95
Type of registered permanent residence		4156		1.20	0.41	1	3
Marital status		4156		0.80	0.40	0	1
Education level		4156		1.22	0.56	1	4
Nationality		4156		0.94	0.25	0	1
Relative income		4156		1.97	0.96	1	5
Number of chronic disease		4156		1.52	1.46	0	8
Smoker or non-smoker		4156		0.17	0.37	0	1
Drinker or non-drinker		4156		0.27	0.44	0	1
Number of leisure activities		4156		1.27	0.67	0	7

**Fig. 1:**
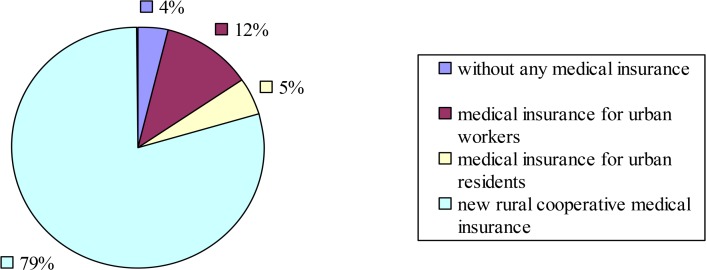
Distribution of medical insurance

Fig. 1 shows the distribution of the elderly that selected different types of medical insurance. Only 4% of the elderly lack any medical insurance, 5% opted for medical insurance for urban residents, and 12% selected the medical insurance for urban employees. The largest percentage was new rural cooperative medical insurance (79%). The options of medical insurance type are ranked from small to large as follows: without medical insurance, medical insurance for urban residents, medical insurance for urban employees, and the new rural cooperative medical system.

**Fig. 2:**
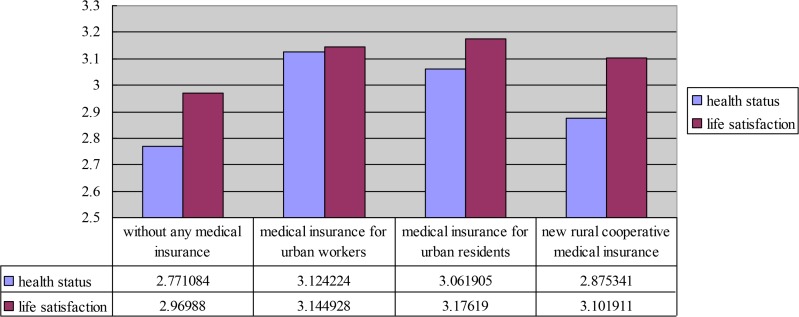
Medical insurance, health status and life satisfaction

Fig. 2 shows the health status and life satisfaction of the elderly with different types of medical insurance. Accordingly, this figure shows the average value of the health status and life satisfaction of the elderly without insurance, with medical insurance for urban residents, and with new rural cooperative medical insurance.

For the relationship of medical insurance and health status, the average value of the elderly who opted for medical insurance for urban employees was larger than that of the elderly who selected medical insurance for urban residents, new rural cooperative medical insurance, and without medical insurance.

The average value of the health status of the elderly without any medical insurance was the lowest. For the relationship of medical insurance and life satisfaction, the average value of the elderly who opted for medical insurance for urban residents was larger than that of the elderly who selected medical insurance for urban employees, new rural cooperative medical insurance, and without medical insurance.

The average value of the life satisfaction of the elderly without medical insurance was the lowest. Therefore, the average value of the elderly who lack medical insurance was the lowest regardless of health status and life satisfaction. The descriptive statistics showed that medical insurance had a significant impact on the health status and life satisfaction of the elderly.

### Research methods

This study focused on the influence of medical insurance on the health and life satisfaction of the elderly. These two indexes were measured using “self-rated health” and “life satisfaction,” and were considered the explained variable. The self-rated health ranged from 1 to 5 (i.e., 1 = very bad, 2 = bad, 3 = average, 4 = good, and 5 = very good). Life satisfaction also ranged from 1 to 5 (i.e., 1 = not satisfied at all, 2 = not too satisfied, 3 = relative satisfied, 4 = very satisfied, and 5 = extremely satisfied).

The two explained variables in this study were discrete ordered variables; thus, the Ordered Probit Model (35) was adopted in this research. The Ordered Probit Model is an extension of the Probit Model, which was designed to process with ordered variables as explained variables. The model was set as follows:

$$Y_{t}=\beta_{t,j}{\mathrm{Insu}}_{t,j}+\sum_{2}^{n}\gamma_{t,i}X_{t,i}+\varepsilon_{t}$$

Where *Y*_1_ is the health status when *t* = 1, *Y*_2_ is the life satisfaction when *t* = 2, and *Insu* is the explanatory variable (i.e., medical insurance). The effects of medical insurance (i.e., medical insurance of urban employees and urban residents and new rural cooperative medical insurance) on health status and life satisfaction were analyzed in this study. Thus, *j* could be 1, 2, and 3; *X_i_* is the controlled variable, and *β_i_* is the coefficient of the corresponding controlled variable.

## Results

### Influence of medical insurance on health status

The health status was considered the explained variable and medical insurance and a few other variables were taken as the independent variable in this study. The Ordered Probit Model, OLS model, and Ordered Logit Model were simultaneously established for regression. Table 3 shows the results. The robustness of the results was compared.

**Table 3:** Effect of medical insurance on health status

**Variable**	**Ordered Probit**	**OLS**	**Ordered Logit**
Medical insurance for urban workers	0.189*(0.110)	0.135**(0.071)	0.319**(0.152)
Medical insurance for urban residents	0.348***(0.121)	0.277***(0.099)	0.604***(0.211)
New rural cooperative medical insurance	0.170**(0.087)	0.145*(0.089)	0.309*(0.192)
Gender	−0.111***(0.042)	−0.098***(0.034)	−0.155**(0.075)
Age (yr)	−0.005*(0.002)	−0.004(0.002)	−0.009*(0.005)
Type of registered permanent residence	0.108(0.069)	0.085(0.056)	0.214*(0.121)
Marital status	0.078*(0.045)	0.063*(0.037)	0.121*(0.079)
Education level	0.033(0.035)	0.027(0.029)	0.057(0.06)
Nationality	0.121*(0.068)	0.103*(0.056)	0.162(0.121)
Relative income	0.155***(0.018)	0.123***(0.015)	0.283***(0.324)
Number of chronic disease	−0.202***(0.012)	−0.163***(0.009)	−0.363***(0.021)
Smoker or non-smoker	−0.013(0.050)	0.009(0.041)	0.029(0.089)
Drinker or non-drinker	−0.090**(0.041)	0.065*(0.034)	0.215***(0.733)
Number of leisure activities	0.127***(0.026)	0.105***(0.021)	0.219***(0.046)

*, ** and *** indicate the significant level are 10%, 5% and 1% respectively.

Table 3 shows that the medical insurance of urban employees and urban residents and the new rural cooperative medical insurance had a significant influence on the health status of the elderly. The regression coefficients were 0.189, 0.348, and 0.170, respectively, thereby indicating that the health status of the elderly with the three medical insurances was better than that of the elderly who lacks health insurance. The health status of the elderly with the medical insurance of urban residents improved more than the elderly who lacks medical insurance. The health status of the elderly who availed of the medical insurance of urban employees ranked second. The health status of the elderly who availed of the new rural cooperative medical insurance improved the least. Moreover, the health status of elderly women was poorer than that of elderly men. The health status of the elderly became increasingly worse with the increase of age. The health status of the elderly with higher income was better than those with lower income. The health status of the elderly who had spouses was better than that of the unmarried elderly. Chronic diseases, as well as smoking and drinking habits, had a negative impact on the health status of the elderly. In addition, having proper leisure activities improves the health of the elderly.

### Influence of medical insurance on life satisfaction

The Ordered Probit Model, OLS model, and Ordered Logit Model were established for regression, with life satisfaction as the explained variable and medical insurance and other variables as the independent variable. Table 4 shows the obtained result.

**Table 4:** Effect of medical insurance on life satisfaction

**Variable**	**Ordered Probit**	**OLS**	**Ordered Logit**
Medical insurance for urban workers	0.236**(0.113)	0.128**(0.061)	0.348**(0.163)
Medical insurance for urban residents	0.307***(0.124)	0.206**(0.084)	0.605***(0.221)
New rural cooperative medical insurance	0.188**(0.090)	0.158**(0.077)	0.452**(0.203)
Gender	0.105**(0.043)	0.074***(0.029)	0.176**(0.078)
Age (yr)	0.003(0.003)	0.002(0.002)	0.005(0.005)
Type of registered permanent residence	−0.013(0.071)	−0.005(0.048)	−0.053(0.127)
Marital status	0.078*(0.046)	0.053*(0.031)	0.155*(0.083)
Education level	−0.023(0.036)	−0.017(0.024)	−0.028(0.065)
Nationality	0.066(0.070)	0.053(0.048)	0.089(0.129)
Relative income	0.037**(0.018)	0.026**(0.013)	0.059*(0.033)
Number of chronic disease	−0.024**(0.012)	−0.015*(0.008)	−0.049**(0.021)
Smoker or non-smoker	−0.075(0.052)	0.054(0.035)	0.115(0.094)
Drinker or non-drinker	−0.069*(0.042)	0.048*(0.029)	0.116*(0.076)
Number of leisure activities	0.046*(0.027)	0.033*(0.018)	0.071*(0.049)

*, ** and *** indicate the significant level are 10%, 5% and 1% respectively.

For the regression result of life satisfaction, Table 4 shows that the medical insurance for urban employees and urban residents and the new rural cooperative medical care had a significant impact on the life satisfaction of the elderly. The regression coefficients were 0.236, 0.307, and 0.188, respectively. These results are significantly positive and indicate that the life satisfaction of the elderly who had medical insurance was higher than that of the elderly who lack any medical insurance. The life satisfaction improvement of urban residents was substantially significant, followed by the urban employees and the elderly who opted for the new rural cooperative medical insurance. The life satisfaction of the elderly who had spouses was higher than that of the elderly who were single. The life satisfaction of the elderly with high income was high. Chronic diseases and drinking habits have negative effects on the life satisfaction of the elderly. Moreover, proper leisure activities increase the life satisfaction of the elderly.

### Robustness test

To test the robustness of the conclusion, two subsamples (i.e., male and female samples) were drawn from the original sample. Among the 4156 sample units, 1506 males and 2650 females accounted for 36.24% and 63.76%, respectively, of the total sample. Other variables were controlled for different subsamples, with the health status and life satisfaction as the explained variables and with medical insurance as the independent variable. The Ordered Probit Model was established for regression. Only the coefficient and significance of the medical insurance of urban employees and urban residents, new rural cooperative medical insurance, and other core variables were presented. Table 5 shows that the self-rated health and life satisfaction models had positive effects on medical insurance, thereby indicating that the conclusion was robust and reliable.

**Table 5:** Robustness test

**Variable**	**Model of self-rated health**		**Model of life satisfaction**	
	**male**	**female**	**male**	**female**
Medical insurance for urban workers	0.154*(0.166)	0.178*(0.103)	0.238*(0.169)	0.165*(0.106)
Medical insurance for urban residents	0.408*(0.232)	0.340**(0.142)	0.146*(0.236)	0.380***(0.147)
New rural cooperative medical insurance	0.091*(0.195)	0.271**(0.139)	0.228*(0.199)	0.242*(0.143)

*, ** and *** indicate the significant level are 10%, 5% and 1% respectively.

## Discussion

The results showed that the medical insurance of urban residents and urban employees and the new rural cooperative medical insurance would improve the health status and life satisfaction of the elderly. The elderly are in their sickly stages, and the ideal medical insurance system and medical security system could keep them healthy and out of poverty, as well as further improve their health status and life satisfaction.

Among the 4156 valid samples, 3297 availed of the new rural cooperative medical insurance (79.33% of the total sample), thereby indicating that this type of insurance has the most number of elderly members compared with the medical insurance of urban employees and urban residents. However, the new rural cooperative medical insurance had a less significant effect on the health status and life satisfaction of the elderly. This result indicates that the popularization and publicity of the new rural cooperative medical service achieved a significant result. However, the ideal degree and security level of the new rural cooperative medical system should be strengthened (36–37).

The self-rated health status and self-rated life satisfaction were considered the explained variables. The disadvantages of the self-rated health status (38–39) and self-rated life satisfaction were their subjectivity. They were based on the individual objective health status and life satisfaction, thereby depending on the individual health status and living conditions of the elderly as the foundation. However, selecting different measurement indexes may lead to different results. Moreover, the ordered model was selected only in comparison with the underlying model but might not be the optimal model. Accordingly, the model would be optimized in the future research.

Several suggestions were proposed that consider the problems determined in this study. First, a universal medical insurance should be continually carried forward, the coverage of medical insurance should be increased, and medical insurance should be promoted from the range, depth, and breadth. Second, the gap between the different security levels of social medical insurance should be decreased when medical insurance is being expanded. The development of a new rural cooperative medical insurance should be prioritized while actively promoting and perfecting the medical insurance of urban residents and urban employees.

## Conclusion

The process of ensuring the healthy physical condition and good life satisfaction of the elderly is a noteworthy problem in the face of severe population aging. The national baseline survey data of the China Health and Retirement Longitudinal Survey in 2013 were adopted in this study. The effects of the medical insurance on health status and life satisfaction of the elderly were empirically analyzed based on the Ordered Probit Model. The results verify the three hypotheses proposed in this study. The basic medical insurance in China, particularly the new rural cooperative medical insurance system, could significantly improve the health status and life satisfaction of the elderly. Thus, establishing and improving a medical insurance system are crucial to improve the health status and life satisfaction of the elderly. Accordingly, introducing a medical insurance is highly significant in establishing equal medical security.

## Ethical considerations

Ethical issues (Including plagiarism, informed consent, misconduct, data fabrication and/or falsification, double publication and/or submission, redundancy, etc.) have been completely observed by the authors.
